# Real space in cryo-EM: the future is local

**DOI:** 10.1107/S2059798321012286

**Published:** 2022-01-25

**Authors:** Colin M. Palmer, Christopher H. S. Aylett

**Affiliations:** a Scientific Computing Department, Science and Technology Facilities Council, Research Complex at Harwell, Didcot OX11 0FA, United Kingdom; b Section for Structural and Synthetic Biology, Department of Infectious Disease, Imperial College London, Imperial College Road, South Kensington, London SW7 2AZ, United Kingdom

**Keywords:** denoising, local resolution, local filtering, noise suppression, real-space measures, real-space filtering, cryo-EM

## Abstract

Cryo-EM images have extremely low signal-to-noise levels because biological macromolecules are highly radiation-sensitive, requiring low-dose imaging, and because the molecules are poor in contrast. Confident recovery of the signal requires the averaging of many images, the iterative optimization of parameters and the introduction of much prior information. Poor parameter estimates, overfitting and variations in signal strength and resolution across the resulting reconstructions remain frequent issues. Because biological samples are real-space phenomena, exhibiting local variations, real-space measures can be both more reliable and more appropriate than Fourier-space measures. Real-space measures can be calculated separately over each differing region of an image or volume. Real-space filters can be applied according to the local need. Powerful prior information, not available in Fourier space, can be introduced in real-space. Priors can be applied in real-space in ways that Fourier space precludes. The treatment of biological phenomena remains highly dependent on spatial frequency, however, which would normally be handled in Fourier space. We believe that measures and filters based around real-space operations on extracted frequency bands, *i*.*e*. a series of band-pass filtered real-space volumes, and over real-space densities of striding (sequentially increasing or decreasing) resolution through Fourier space are the best way to address this and will perform better than global Fourier-space-based approaches. Future developments in image processing within the field are generally expected to be based on a mixture of both rationally designed and deep-learning approaches, and to incorporate novel prior information from developments such as *AlphaFold*. Regardless of approach, it is clear that ‘locality’, through real-space measures, filters and processing, will become central to image processing.

## 1 Cryogenic electron microscopy (cryo-EM) yields exceptionally noisy, unreliable images of the electron scattering density, from which information is recovered by averaging

Imaging of biological samples is usually limited by the effects of radiation on the sample. Radiation damage occurs quickly in macromolecules since they are radiation-soft and rely on weak forces for their stability (Glaeser, 1971; Knapek & Dubochet, 1980). Richard Henderson established that electrons were the best practical form of radiation to use for the imaging of biomolecules in terms of the amount of information gained before substantial radiation damage occurs, provided that the sample can be prepared as a thin film in vacuum, in order to avoid multiple scattering events, and can be considered a weak-phase object for elastic scattering (Henderson, 1995; Peet *et al*., 2019). Direct electron detectors are approaching near-perfect electron imaging, in which the impacts of essentially all electrons are recorded and well localized at the detector (Li *et al*., 2013; McMullan *et al*., 2014).

Even when using electrons and detecting them with high accuracy, however, the dose that can be tolerated before the sample is destroyed is tiny, leading to very imperfect images. This low dose translates directly into a low signal-to-noise ratio (SNR). The principal contributor to this is shot noise because, while the scattering probability of electrons is best described as a waveform, the electrons that contribute to the image are detected as individual particles (Baxter *et al*., 2009). Radiation damage from inelastic scattering is also a major contributor to the poor SNR, accumulating over the course of an exposure and contaminating the signal from earlier frames. In order to recover the desired information from low-SNR images it is necessary to average similar samples together (Frank *et al*., 1981). Because the stochastic noise is zero-mean in Fourier space, the average will tend to the original signal given correction for the contrast transfer function (CTF; Zemlin, 1978).

## 2 The low SNR of cryo-EM images substantially hinders parameter estimation and thereby all further reconstruction steps

If high-SNR images could be recorded, every step in cryo-EM image processing would be effectively trivial. Even for low-SNR images, with appropriate correction of the CTF (Zemlin, 1978) averaging of correctly weighted particle images in the correct orientation should yield structures to the limit of the information present in the data (Frank *et al*., 1981). However, at low SNR the estimation of these parameters is fraught with difficulty and error, and the effects of low SNR on parameter estimation therefore represent the fundamental issue in cryo-EM (van Heel, 1987; Radermacher *et al*., 1987; Penczek *et al*., 1994). These difficulties are exacerbated by the number of parameters requiring estimation. During image collection there is frequency-dependent radiation damage (Grant & Grigorieff, 2015) and three-dimensional movement of the sample, the two in-plane dimensions of which are represented as offsets (Li *et al*., 2013) and the third of which is incorporated into the contrast transfer function, which represents a further three parameters (Mindell & Grigorieff, 2003). For each image or particle, three angles and two offsets relative to the object of interest must be calculated [which are estimated differently for tomography, subtomogram averaging and single-particle analysis (SPA)]. Finally, the spectral SNR curve of the objects is required for deconvolution of the CTF from the sample through Wiener filtering or a similar process (Wiener, 1942; Kirkland *et al*., 1980; Scheres, 2012a).

## 3 Noise and uncertainty in cryo-EM has traditionally been handled globally as a ‘resolution’ and a concomitant global low-pass filter

Inaccurate parameter estimation results in substantial noise error in any reconstruction from cryo-EM. Because reconstruction of a three-dimensional volume from two-dimensional images is an ill-posed problem, it is typically handled by a regularized, iterative process seeking a local minimum. In such processes output from the previous iteration is used as input for the next, and incorporation of noise is highly problematic. Any noise that is retained will become part of the target in subsequent iterations, indistinguishable from the signal and thereby becoming fixed in the resulting reconstructions. This problem is referred to as ‘overfitting’. The diagnosis of such issues requires some way to separate the signal from the noise, and therefore the practice of splitting data into ‘half sets’ has been adopted, making reconstructions from each half of a data set and comparing the two to estimate their similarities and differences. To avoid overfitting, these reconstructions must remain truly independent, with the entire parameter estimation and reconstruction process performed separately for each set (Grigorieff, 2000). The standard way to measure the similarity between the half-set reconstructions is with the Fourier shell correlation (FSC), which is calculated in Fourier space and therefore represents a weighted average of the estimate of the SNR over the entire volume in each resolution shell (Harauz & van Heel, 1986). As an indicator of interpretability, a resolution value for the reconstruction is typically assigned based on the point at which the FSC curve drops below a certain threshold value. [A figure of 0.143 is often used for this threshold, although the details have remained somewhat controversial. A detailed consideration of these problems as they apply to locality can be found in Rohou (2020).] Usually, the final reconstruction is low-pass filtered at the assigned resolution, either simply with a standard fall-off, or in a more sophisticated advance, through a global weighting based on the FSC curve itself (Rosenthal & Henderson, 2003).

## 4 The samples most commonly investigated by cryo-EM typically exhibit large local variations in signal in three dimensions

For independent cryo-EM micrographs, the SNR within the field of view tends to be relatively similar throughout. Variations in ice thickness and sample scattering density result in local differences; however, the process of image generation is similar unless there is substantial variation in defocus across the field of view, such as when a tilt has been applied during tomography. The specimens investigated by cryo-EM, however, are unrestrained, flexible and frequently dissimilar at a structural level. They range from unique assemblages such as cells and organelles, which cannot be averaged as whole bodies, through partially similar assemblies such as focal adhesion complexes and cytoskeletal elements, which will have substantial compositional variation, to well ordered and homogenous individual particles. Even when the chemical composition of a series of samples is identical, there will still be some degree of conformational heterogeneity, and their three-dimensional structures will vary. As a result of this variation, when such particles are averaged together those regions with higher similarity will produce a stronger signal, while less similar regions will have a weaker signal and comparatively higher noise levels.

## 5 Global assessment and filtering in Fourier space cannot correctly account for local variations within the sample

The requirement to average low-SNR images of structurally different samples to generate a cryo-EM reconstruction implies that the output, a single structure, will not be an accurate three-dimensional representation. All regions of variation will be represented as an average of the input conformers or compositions. Moreover, as explained above, any volumes resulting from averaging will exhibit differences in local SNR in their different regions. Because both signal and noise in cryo-EM are ‘coloured’, *i*.*e*. they vary with frequency, resolution-based methods are essential to separate high-fidelity from low-fidelity features. Assessment of the reconstruction in Fourier space (for example calculation of the FSC curve) cannot account for local variations in real space. Fourier-space approaches to filtering and weighting of cryo-EM reconstructions (such as the FSC weighting discussed above) inherently have a global effect in real space, affecting all local regions equally. However, because the SNR varies between regions, global filtering is inappropriate, leading to excessive blurring of high-resolution regions and over-sharpening of noisy low-resolution regions. Therefore, both global assessment and global weighting of cryo-EM densities are problematic; they can be misleading and in many cases harmful to the final reconstruction. It remains essential to retain the resolution-based aspects of Fourier-based methods; however, real-space variations cannot be ignored.

## 6 Several methods of achieving locality for Fourier space have been defined; however, real-space measures and operations on extracted frequency bands are superior in performance

**Figure 1:**
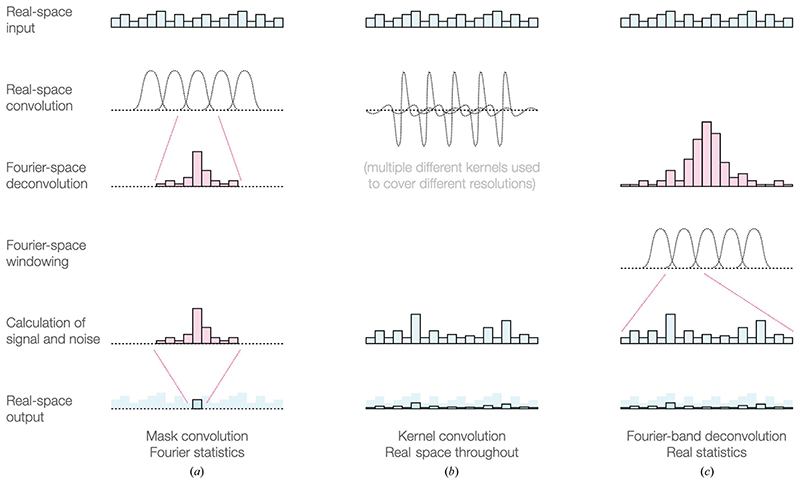
Three approaches to local evaluation and filtering: real-space windowing with Fourier evaluation, real-space kernel convolution and Fourier-shell real-space mapping. (*a*) Convolution of a real-space window, followed by Fourier transformation and evaluation or filtering. (*b*) Convolution of a series of real-space kernels corresponding to a basis for evaluation and/or filtration. (*c*) Generation of real-space maps from individual Fourier bands, rings or shells, followed by evaluation or final summation. We believe this method to be the most promising approach.

It would be natural to handle local variations by working directly in real space. However, many important phenomena in cryo-EM, including signal and noise strength, radiation damage and the CTF of the microscope, are directly dependent on spatial frequency. It is much easier to account for these in Fourier space, and so global Fourier-space approaches have generally been preferred. Accurate treatment of cryo-EM images requires working in a way that is both local and frequency-dependent. This is not trivial, and several different approaches have been tried (Fig. 1). For a long time, experimenters in SPA have achieved a degree of locality by using a binary mask to separate the particle from the surrounding solvent and restricting the calculation of the FSC to the particle alone. Cardone *et al*. (2013) extended this approach to measure resolution locally by convolving a mask with each point of the reconstructed volume (Fig. 1a). At each point in the map, the mask is applied to both half-maps, the respective Fourier transforms are calculated and the resolution is measured using the standard FSC curve method.

This achieves locality well, but is extremely computationally intensive because it requires Fourier transforms to be calculated for each point, and despite the name fast Fourier transformation is not so fast when calculated thousands of times. Furthermore, the results become highly dependent on the properties of the mask, and the statistics are hampered by the relatively small number of pixels or voxels within each region (Cardone *et al*., 2013; Chen *et al*., 2013). A computationally more tractable approach was adopted through the use of real-space kernels allowing the estimation of components at each resolution (Kucukelbir *et al*., 2014; Fig. 1b); however, preprocessing and the relative sharpness of the map have large effects on the output of these approaches, making the results somewhat inconsistent and unreliable.

The most promising approach to achieving locality is the application of band-pass filters and subsequent transformation of each band into real space for operations, or equivalently consideration of the real-space differences between successive low-pass filtered densities (Vilas *et al*., 2018; Ramlaul *et al*., 2019; Ramlaul, Palmer *et al*., 2020; Penczek, 2020). This has the benefit of being more computationally tractable than mask-convolution approaches, because Fourier transforms only need to be calculated at each frequency considered rather than for each real-space point. It also avoids spurious effects of the mask size and shape, and allows direct comparison of densities over the entire map, but retains independence from the effects of sharpening or differences in power between frequency shells that are so problematic for purely real-space kernel-convolution-based approaches. Furthermore, parameter estimates from this approach can often be better than standard estimates in Fourier space for a number of reasons. Firstly, the number of data points (in real space) available for calculations at each frequency is much larger, simplifying the estimation of distributions, and is consistent between frequency bands, facilitating direct comparisons between parameters estimated at different resolutions. Secondly, direct comparisons between Fourier transforms require working in a four-dimensional space because the Fourier coefficients are complex numbers, whereas real space limits the joint distribution to two dimensions. Finally, working in narrow frequency bands avoids the large (logarithmic) variations in signal power which are a natural consequence of working in Fourier space. One last advantage of this approach is that it can be applied at arbitrarily high (subpixel) resolution in either Fourier space or real space. This allows the smooth treatment of, for example, real-space differences within a tilted micrograph at the correct defocus, or equiphase bands of Fourier voxels according to the phase of the CTF through padding.

There are, of course, constraints upon the resolution that can be achieved in Fourier space when one is converting to real space in this way, although it is important to note that these apply to any method of attaining locality involving Fourier space. While the real-space representation of any resolution-based calculation can be considered as a simple summation of waveforms, allowing appropriate weighting to be performed in either space, the behaviour of such waveform summations can be counterintuitive, especially at discontinuities in either space. A widely known example of this in real space is Gibbs’ phenomenon, whereby the partial summation of a Fourier series exhibits an overshoot at jump discontinuities. While biological macromolecules can generally be considered to exhibit smooth, continuously differentiable, densities, such phenomena limit how narrow the bandpass or low-pass filters in use may be, as such an overshoot or ringing will make the real-space values unrepresentative for further calculations.

## 7 Acceptance of these issues as they apply to resolution calculation necessitated the beginning of a real-space ‘local’ perspective in cryo-EM

The identification of real-space problems in SPA led to the definition of ‘local’ resolution maps (Cardone *et al*., 2013; Kucukelbir *et al*., 2014), which defined a similar approach to the global FSC cutoff and filtering previously established for reconstructions. Within each region of a map, a resolution cutoff figure is ascribed, providing a baseline for interpretation. There are several issues with this approach, some of which are inherited directly from approaches to global resolution measurement. Firstly, the choice of which resolution threshold to use remains contested. In smaller regions of the map, the number of samples available is much reduced and so such locally calculated statistics are inherently more noisy and variable. Furthermore, a single resolution figure does not provide a secure foundation for interpretation; the complete shape of the spectral SNR curve is required for a meaningful understanding of the reliability of features in real space.

## 8 Defining local resolution is insufficient for proper interpretation and processing; local filtering or density modification is essential for the effective use of such information

Measurement of local SNR variation gives some insight into the quality of a reconstruction, but it is of limited usefulness without treatment. In spite of knowledge of the reliability, or lack thereof, in each region, it is difficult for an experimenter to visualize such issues when interpreting their density. Furthermore, for further computational processing, including for further parameter estimation, it is essential to account for these local SNR variations or overfitting will occur. This issue is obvious during interpretation by the scientist and has been shown to be a substantial issue in the refinement of particle orientations (Punjani *et al*., 2020; Ramlaul, Palmer *et al*., 2020). As well as improving the map reconstruction itself, the appropriate treatment of local SNR variations can improve the results of other key processing steps in cryo-EM [*e*.*g*. particle picking, see Bepler *et al*. (2020)].

## 9 Different approaches are required when filtering for optimal signal recovery rather than interpretation

**Figure 2:**
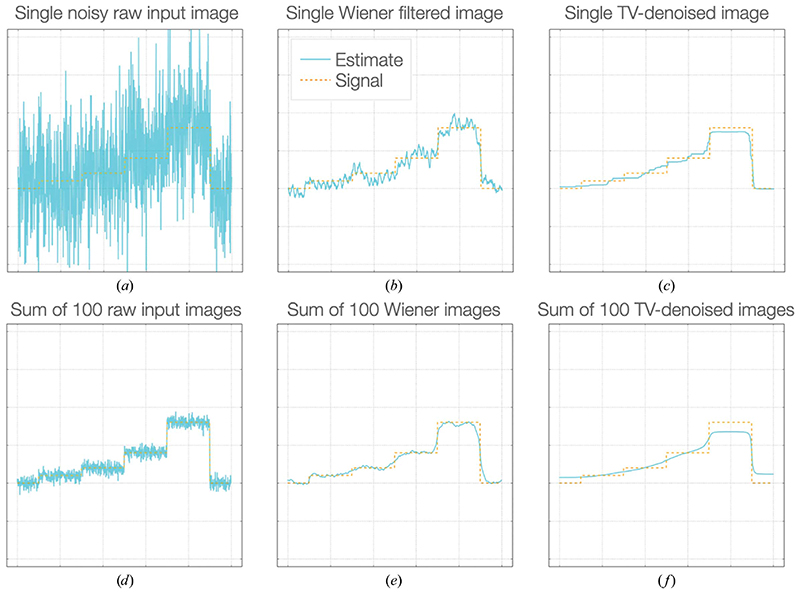
A comparison of linear noise-suppression and nonlinear denoising approaches for local filtering. (*a*) A set of noisy input images were generated (blue line; true signal, dashed orange line). A noise-suppression approach (Wiener filtration: weighting according to the SNR in Fourier space) reduces the noise (*b*), whereas a denoising approach (total variation regularization: optimization to minimize the magnitude of the gradient while retaining maximum fidelity) removes much more visible noise and flattens spurious features (*c*). On averaging, the raw images (*d*) and Wiener filtered images (*e*) tend towards the true signal, however, the denoised images (*f*) do not. Therefore, if images are to be used for further averaging, only a linear weighting, noise-suppression approach is appropriate.

Because of the low-SNR aspects of local filtering, it is important to apply different targets for filtering intended for downstream averaging in comparison to filtering for down-stream interpretation or for use as prior information in processing (Fig. 2). For averaging, ‘noise suppression’ is required; in such cases it is best to weight the input according to an accurately estimated SNR for maximum information recovery. Further averaging of such images or volumes benefits from accounting for the local SNR and therefore ‘Wiener’-like weighting is the most appropriate target. However, for downstream interpretation or use as a prior, full ‘denoising’ is more apt; in such cases one wishes to exclude as much noise as possible to prevent overfitting caused by the stable incorporation of noise into models or parameters. Probabilistic weighting according to significance is most sensible in these cases (Beckers *et al*., 2019; Ramlaul *et al*., 2019), although a wide variety of denoising algorithms are available. It is important to note that data filtered in such a manner should no longer be averaged, as the weighting will not be linear with the signal.

## 10 Mixed real- and Fourier-space techniques have only recently begun to allow the proper decomposition of conformational heterogeneity

Ideally, rather than averaging over differences, one would prefer to account for them in the reconstruction process. Compositional heterogeneity is ideally resolved by sorting particles or subtomograms into complexes of different chemical composition. This works well in the case of the presence or absence of large macromolecules, but is problematic for small ligands or side chains exhibiting radiation damage. Conformational flexibility, on the other hand, is a key feature of most macromolecules, often representing the *raison d’être* for molecular machines, and is a rather more important contributor to local variation in SNR than compositional variations. By its nature it can only be resolved in real space. Fundamentally, however, every macromolecule imaged is likely to lie in a class of one: differences will remain, the only variable is their scale and significance, and therefore local filtering will remain key. Current techniques to handle flexibility rely on either the deconvolution of the sample into different rigid regions with separate parametrizations [as multiple bodies, for example, *RELION* multibody refinement (Nakane *et al*., 2018), or through serial subtraction] or through the computational generation of manifolds of greatly reduced dimensionality onto which the different particles can be placed (Frank & Ourmazd, 2016; Zhong *et al*., 2020; Punjani & Fleet, 2021b). In the case of separate parametrizations, this is functionally equivalent to performing multiple, restrained independent reconstructions of subregions of the sample in question. The manifold approach, on the other hand, is relational, matching particles or subtomograms to a position within a continuum of structures. It provides more information on the motions resolved, but typically attains lower overall resolution because information is spread over a continuum. Recently, however, the combination of deep learning with the introduction of real-space prior information has begun to allow per-object treatment of flexibility for the first time (Punjani & Fleet, 2021*a*).

## 11 Introducing prior knowledge is trivial in real space but is very difficult in Fourier space

**Figure 3:**
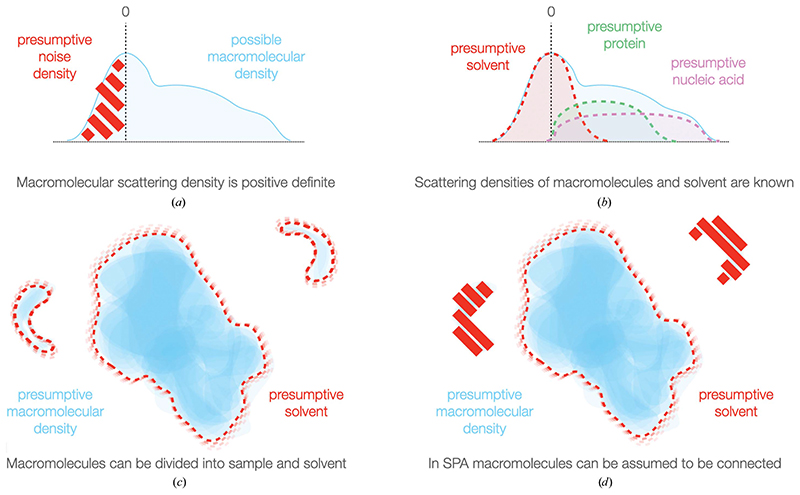
Incorporation of prior information in real space. (*a*) Macromolecular density is positive definite, which provides a substantial restraint. (*b*) The densities of protein and nucleic acids are known, and such information can be incorporated into cryo-EM real-space processing as a real-space prior. (*c*) Macromolecules are composed of either sample or solvent, allowing binary restraints leading on from existing masking approaches. (*d*) For singleparticle analysis, connectivity can be assumed and provides a further restraint, which is already partially utilized in real space.

Because reconstruction is an ill-posed problem, restraints are usually incorporated in Fourier space to regularize the refinement process. However, the available prior information that can be readily incorporated in Fourier space is extremely limited: either ‘smoothness’ or an explicit Gaussian prior are the typical restraints implemented in current software (Scheres, 2012*b*; Punjani *et al*., 2017). In real space a large number of other restrictions on the nature of the density can be implemented as constraints (Fig. 3). Many have been of great utility in crystallographic reconstruction and have equal potential in cryo-EM. Macromolecular densities are connected, can be divided into a binary solvent and macromolecule regions, are effectively positive definite against a solvent background, exhibit a reliable scattering density difference from solvent and typically have an expected scattering mass, all of which can be incorporated into restraints (reviewed in Podjarny *et al*., 1996; Jakobi *et al*., 2017; Terwilliger *et al*., 2020). The use of such restraints based on the simple division of particles into solvent and sample of constrained density has allowed the first treatment of conformational flexibility by Punjani & Fleet (2021*a*). Using coarse-to-fine low-pass resolution extension, they were able to train a neural network to generate 3D deformation fields best matching an input structure to flexible particles. Optimization of this ‘canonical’ volume in real space then partially resolves the flexibility of the particles, recovering significantly higher resolution than the input.

The incorporation of further prior knowledge about macromolecular features from known protein structures has also been suggested and has great potential. However, the incorporation of such rich priors remains very difficult for unknown structures, as this information is directly used for interpretation and evaluation of the final density. There is a substantial risk of severe overfitting because the prior alone might produce realistic-looking features (Kimanius *et al*., 2021). A promising alternative that has long been used in crystallography, and for which validation methodologies have been developed (Liebschner *et al*., 2019; Brown *et al*., 2015), is the incorporation of information from partial and ensemble structures. The success of *AlphaFold* (Jumper *et al*., 2021) has rendered such input priors readily available. The ideal model would be a three-dimensional structure of the individual molecule(s) that can be deformed to fit each separate observation through, for example, molecular-dynamics simulations or neural networks.

## 12 Future image-processing pipelines for isolated macromolecules and in situ studies will converge, but both must absorb local techniques

It seems highly likely that the SPA and subtomogram averaging approaches will converge. Subtomogram refinement has already adopted the techniques of SPA in order to reach high resolution. SPA will similarly require techniques coming from tomography to handle flexibility and variation in sample height across the field of view, and to work effectively within cells and with complex samples. A key requirement for progress will be a substantial reduction in the number of images that are required for a tomographic reconstruction, because this is often limiting and engineering avenues to address this are limited. At least two different images of each individual object under consideration will of course be required to resolve the relative positions of components in Z for averaging; however, localization of objects may well prove to be possible with many fewer images than are currently collected, allowing more of the dose and imaging time to be expended on useful imaging. This is possible both through advances in the identification of components in projections alone and in the low-resolution reconstruction of individual tomograms from fewer images. Compressed sensing techniques, in which structures are parametrized on a sparse basis and L1-norm regularization is used to resolve ambiguities and select the ‘simplest’ among numerous possible structures, appear to have substantial promise for such advances. This will allow the use of fewer observations and under-determined reconstruction systems, and can be seen as a form of formalized Occam’s razor (Candès *et al*., 2006; Ockham, c.1330).

It is generally accepted that future image-processing development will be driven by a mixture of classical approaches and novel deep-learning algorithms. Classical approaches no longer represent the cutting edge of what can be achieved with computational techniques and advances are often too computationally intensive for ready utility, whereas deep-learning techniques bring challenges for validation and generalization, since it is difficult to establish what prior information is being used and how reliable the resulting structures are. Whatever the underlying approach, however, we believe that real-space processing of image segments isolated in Fourier space, and local approaches more generally, will become central themes. Global Fourier-space approaches are insufficient to handle the problems inherent in the averaging of cryo-EM data, cannot readily incorporate the necessary prior information, which is comparatively trivial in real space, and cannot adequately account for flexibility or heterogeneity as these are real-space phenomena.
